# Gluten-free diet exposure prohibits pathobiont expansion and gluten sensitive enteropathy in B cell deficient J_H_^-/-^ mice

**DOI:** 10.1371/journal.pone.0264977

**Published:** 2022-03-24

**Authors:** Ahmed Dawood Mohammed, Nia Hall, Ioulia Chatzistamou, Amy Jolly, Jason Lee Kubinak

**Affiliations:** Department of Pathology, Microbiology, and Immunology, University of South Carolina School of Medicine, Columbia, SC, United States of America; Toho University Graduate School of Medicine, JAPAN

## Abstract

In humans, celiac disease (CeD) is a T-cell-driven gluten-sensitive enteropathy (GSE) localized to the small bowel (duodenum). The presence of antibodies specific for gluten- and self-antigens are commonly used diagnostic biomarkers of CeD and are considered to play a role in GSE pathogenesis. Previously, we have described an apparent T-cell-mediated GSE in CD19^-/-^ mice, which develop weak and abnormal B cell responses. Here, we expand on this observation and use a mouse model of complete B cell deficiency (J_H_^-/-^ mice), to show that absence of a humoral immune response also promotes development of a GSE. Furthermore, 16S analysis of microbial communities in the small intestine demonstrates that a gluten-free diet suppresses the expansion of anaerobic bacteria in the small intestine and colonization of the small intestine by a specific pathobiont. Finally, we also observe that SI enteropathy in mice fed a gluten-rich diet is positively correlated with the abundance of several microbial peptidase genes, which supports that bacterial metabolism of gluten may be an important driver of GSE in our model. Collectively, results from our experiments indicate that J_H_^-/-^ mice will be a useful resource to investigators seeking to empirically delineate the contribution of humoral immunity on GSE pathogenesis, and support the hypothesis that humoral immunity promotes tolerance to gluten.

## Introduction

The term "gluten" refers to a complex mixture of several storage proteins found in wheat and other cereal grains [1]. Upon ingestion, host enzymes, as well as enzymes encoded by bacteria residing in the gut, can modify the chemical structure of gluten [2–4], which in the latter case has been exploited in the food industry to promote a more palatable texture to cereal-based foods. However, host and bacterial modifications to the structure of gluten can also make it more immunogenic [2,5], which can lead to the development of "gluten sensitivity"; a general term that refers to the development of an abnormal immune response to gluten antigens. Gluten sensitivity is increasing in incidence globally [6]. CeD, as well as non-celiac gluten sensitivity (NCGS), are the two classes of gluten sensitivity that differ in clinical presentation and underlying genetics [7]. CeD is an immune-mediated enteropathy characterized by villous blunting and crypt hyperplasia that is strongly linked to the presence of polymorphisms in the loci encoding specific HLA-DQ heterodimers (DQ2 and DQ8) [7]. Indeed, in a recent meta-analysis, approximately 95% of all CeD patients were shown to possess at least one copy of the HLA-DQB1*02 allele that encodes the β chain of the DQ2 heterodimer [8]. In contrast, NCGS is characterized by a symptomatic response to gluten ingestion that does not result in villous blunting and crypt hyperplasia and is not associated with specific HLA risk alleles [9].

The GSE observed in CeD patients is a T-cell-mediated pathology [10] and antigen presentation by B cells can promote the activation of gluten-specific T cells [11]. However, what is less clear is a role for antibodies in GSE pathogenesis. This is an important and fundamental question because despite the identification of tissue transglutaminase (tTG) as a major autoantigen targeted by antibodies in CeD [12] (the basis for why it is widely labelled an autoimmune disorder), no study to date has demonstrated that anti-tTG antibodies are pathogenic. Moreover, antibodies generated against immunogenic gliadin subunits of gluten (anti-gliadin antibodies (AGAs)) are also enriched in CeD patients, are commonly used as a biomarker of gluten sensitivity, but have also never been shown to drive GSE pathogenesis [13]. Ironically, antibody deficiency (specifically immunoglobulin A (IgA) deficiency) is a commonly observed comorbidity in CeD patients, with the incidence of IgA deficiency estimated to be 10–15 fold higher in CeD patients [14].

Despite the strong link between specific HLA-DQ alleles and CeD, only a minority of people carrying these risk alleles will go on to develop CeD, which implies that other factors contribute to CeD pathogenesis [8]. Alterations to the gut microbiota has been postulated to be a potentially important environmental trigger, and several studies have demonstrated a link between CeD and abnormal gut microbial ecology (reviewed in [15]). Through the production of secretory immunoglobulin A (IgA), the mucosal humoral immune response has been shown to play an important role in regulating gut microbiota composition [16–22] and function [23,24]. However, the incidence of IgA deficiency has been estimated to be 10–15 times higher in CeD patients [14]. Thus, it is possible that antibody deficiency could enhance susceptibility to GSE in genetically prone individuals by resulting in the development of an abnormal microbiota that enhances gluten sensitivity.

Previously, our group has demonstrated that CD19^-/-^ mice, which exhibit weaker and abnormal humoral immune responses, developed a chronic SI enteropathy localized to the ileum of CD19^-/-^ mice. This SI-specific enteropathy was associated with outgrowth of anaerobic bacteria in the SI, the development of a metabolic syndrome characterized by lipid malabsorption and enhanced innate and adaptive (T-cell-mediated) immune responses [25]. More importantly, we determined this phenotype to be a microbiota-dependent and gluten-sensitive enteropathy as administration of antibiotics or a gluten-free diet protected animals from disease. Here, we have extended upon these observations, to demonstrate in an independent model of complete B-cell-deficiency (J_H_^-/-^ mice), that the lack of a humoral immune response is associated with development of SI enteropathy that can be rescued by administration of a gluten-free diet. Additionally, we have performed 16S rRNA gene sequencing in an effort to identify relevant microbial phenotypes that are responsive to the presence/absence of dietary gluten in an effort to identify putative mechanisms by which dysbiosis may contribute to the pathogenesis of GSE. Collectively, results from this study and previous observations mad in CD19^-/-^ mice suggest that an intact humoral immune response may be important for tolerizing individuals to dietary gluten. We provide a critical review of our results within the context of results derived from other mouse models of GSE as well as human studies.

## Materials and methods

### Mice

Male and female mice from each of the mouse strains utilized in this study were purchased from Jackson Laboratories (WT C57BL/6 (catalog#000664); J_H_^-/-^ (catalog#002438); CD19^-/-^ (catalog#006785); RAG1^-/-^ (catalog#002216)) and were used to establish a long-term breeding colony at the University of South Carolina. All animals used in this study were derived from this breeding colony. All mouse strains possess a C57BL/6 genetic background. Animals were reared and maintained under identical SPF conditions in a single environmentally-controlled room exclusively used to house this mouse colony. All animals were maintained under constant environmental conditions (70°F, 50% relative humidity, 12:12 light:dark cycles) and were given *ad libitum* access to autoclaved drinking water and an irradiated mouse chow containing gluten (Envigo; diet#2920X). Prior to diet exposure, male and female mice derived from the same litters were randomly assigned to diet treatment. During diet exposure, mice were singly-housed to avoid the confounding effect of co-housing coprophagic animals on microbiota composition. At the completion of diet exposure experiments, mice were euthanized for sample collection. All experimental mice were between the ages of 8 and 16 weeks of age at time of sacrifice.

### Ethics statement

All animal use strictly adhered to federal regulations and guidelines set forth by the University of South Carolina Institutional Animal Care and Use Committee (IACUC)(IACUC-approved protocol#101580). Animal sacrifice was part of this study with CO_2_ asphyxiation as the primary means of euthanasia and cervical dislocation as the secondary means of assurance.

### Diets and experimental design

Four independent experimental replicates were performed for this study; each utilizing a different source of gluten-rich diet (GRD) and gluten-free diet (GFD). All GFDs and GRDs incorporated either casein or gluten as the dominant dietary protein, respectively. Replicate #1 consisted of exposing J_H_^-/-^ mice (derived from homozygote J_H_^-/-^ x J_H_^-/-^ crosses) to a purified GFD (AIN-76A)(BioServ cat#F1515) or maintaining them on the GRD on which they had already been maintained on prior to commencement of the study (2920X diet). Animals were maintained on these diets for eight weeks. Replicate #2 consisted of exposing J_H_^-/-^ mice (derived from homozygote J_H_^-/-^ x J_H_^-/-^ crosses) to the purified GFD (AIN-76A) or a distinct GRD (AIN-93G-(gluten))(BioServ cat#S7784). These mice were maintained on this diet for three weeks. Replicate #3 consisted of exposing J_H_^-/-^ mice (derived from J_H_^+/-^ x J_H_^+/-^ heterozygote crosses) to the same diets as replicate #2 animals, and animals were also maintained on these diets for three weeks. Replicate #4 consisted of exposing J_H_^-/-^ mice (derived from homozygote J_H_^-/-^ x J_H_^-/-^ crosses) to the GRD (AIN-93G) or a completely nutritionally-matched GFD (AIN-93G-(casein))(BioServ cat#F3156). Animals were maintained on this diet for eight weeks. Animals were given ad libitum access to these respective diets for the duration of the experiments with food being replaced weekly. The compositional breakdown of each diet is freely available and can each be found on vendor websites.

### Enteropathy scoring

Enteropathy scoring was performed by a blinded pathologist using a modified version of the Marsh scoring method as previously described [25].

### 16S rRNA gene sequencing

Fecal and SI (ileal) luminal contents were collected from euthanized mice and frozen at -80°C until downstream processing. DNA was isolated from samples using the QIAamp DNA MIcrobiome Kit (Qiagen, cat#51704) that included a 3 minute bead-beating step. Isolated DNA was submitted to the University of Alabama Heflin Center Genomics Core for paired-end 16S rRNA gene sequencing on an Illumina MiSeq instrument. Raw fastq files were demultiplexed, with forward and reverse primer sequences trimmed from the reads. This yielded high quality (QC score>15) 251bp sequence amplicons spanning the V3/V4 region of the bacterial 16S rRNA gene. Demultiplexed fastq files were then imported into QIIME 2.0 for downstream quality-filtering and sequence analysis. Data denoising was performed using the DADA2 algorithm to remove low quality reads and chimeric sequences. All samples were rarified to an even sampling depth of 17,649 sequences prior to conducting α and β analyses, which adequately captures most of the amplicon sequence (ASV) diversity across samples (S1 Fig). For taxonomic analyses, a custom classifier based on our own dataset was trained against the Greengenes 13.8 database at a 99% ASV sequence identity threshold.

### Statistics

**Fig 1:**
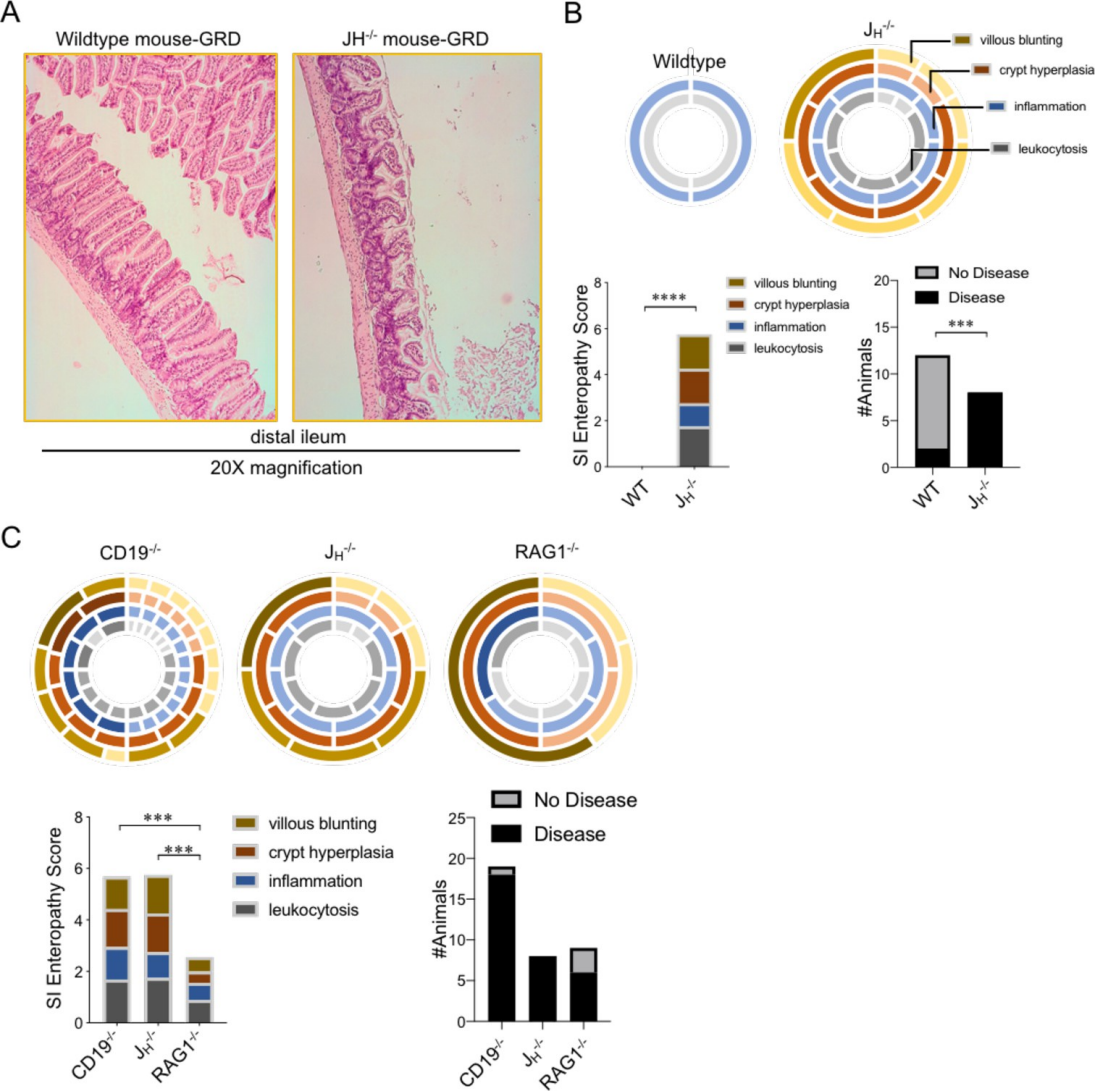
J_H_^-/-^ mice develop a gluten-sensitive SI enteropathy, which is a T cell-dependent phenotype. **(A)** Representative H&E staining of WT and J_H_^-/-^ mouse ileums are shown. **(B)** SI enteropathy scores and the incidence of detectable disease phenotypes are shown. The wheal diagrams depict the severity of disease (darker = more severe) for each of the four disease phenotypes (outlined in plot). Spokes (i.e. breaks) in the wheel denote the number of mice where a given phenotype was observed. **(C)** SI enteropathy scores and disease incidence comparisons are shown for J_H_^-/-^, CD19^-/-^, and RAG1^-/-^ mice. (B and C) Two-way repeated measure ANOVAs were used to compare the effects of genotype on the severity of SI enteropathy, and a two-sided Fisher’s exact test was used to compare disease incidence, in both B and C.

**Fig 2:**
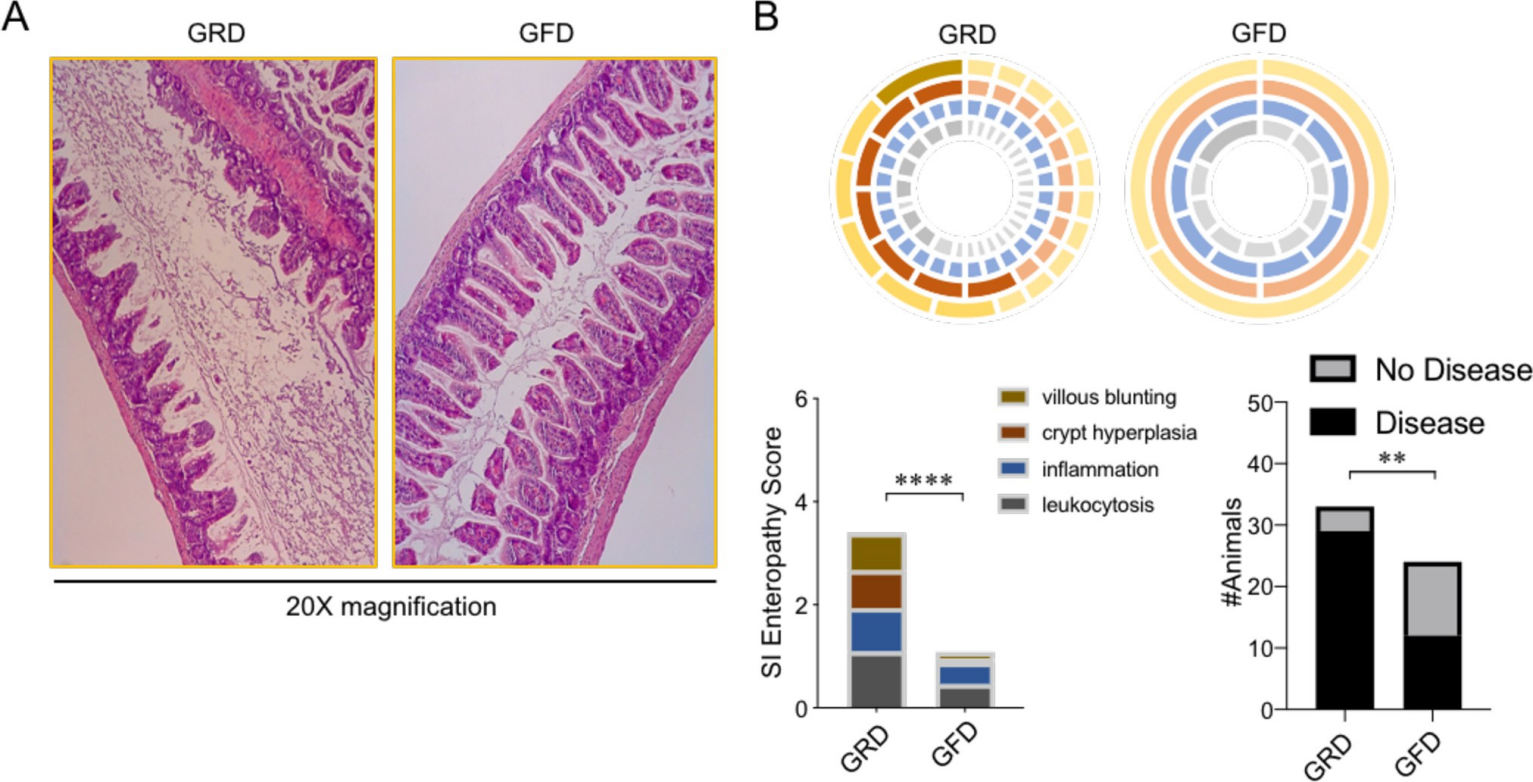
Exposure to a gluten-free diet ameliorates the severity of enteropathy in JH^-/-^ mice. **(A)** Representative H&E staining of J_H_^-/-^ mouse ileums from animals that underwent GRD or GFD exposure are shown. **(B)** SI enteropathy scores and the incidence of detectable disease phenotypes are shown. (B) Two-way repeated measure ANOVAs were used to compare the effects of genotype on the severity of SI enteropathy, and a two-sided Fisher’s exact test was used to compare disease incidence.

**Fig 3:**
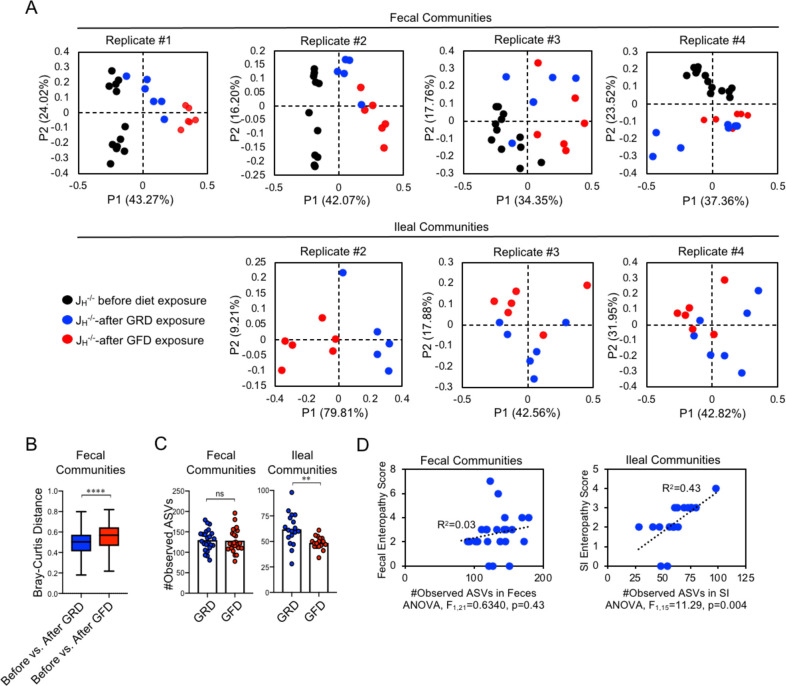
A GFD alters microbiota composition in JH^-/-^ mice. **(A)** PcoA plots based on Bray-Curtis (BC) analysis of fecal and SI microbial communities across replicate experiments are shown. P-values in plots denote results of PERMANOVA hypothesis testing. **(B)** Between-group BC distances, reflecting the extent of divergence in microbiota composition before and after diet exposure, are shown. Mann-Whitney U test. **(C)** The number of observed species (ASVs) is shown between GRD and GFD J_H_^-/-^ cohorts. Student’s t-test; ns = non-significant, ** = p<0.01. **(D)** Results if linear regression analysis of the relationship between species diversity and SI enteropathy scores are shown. One-way ANOVA.

**Fig 4:**
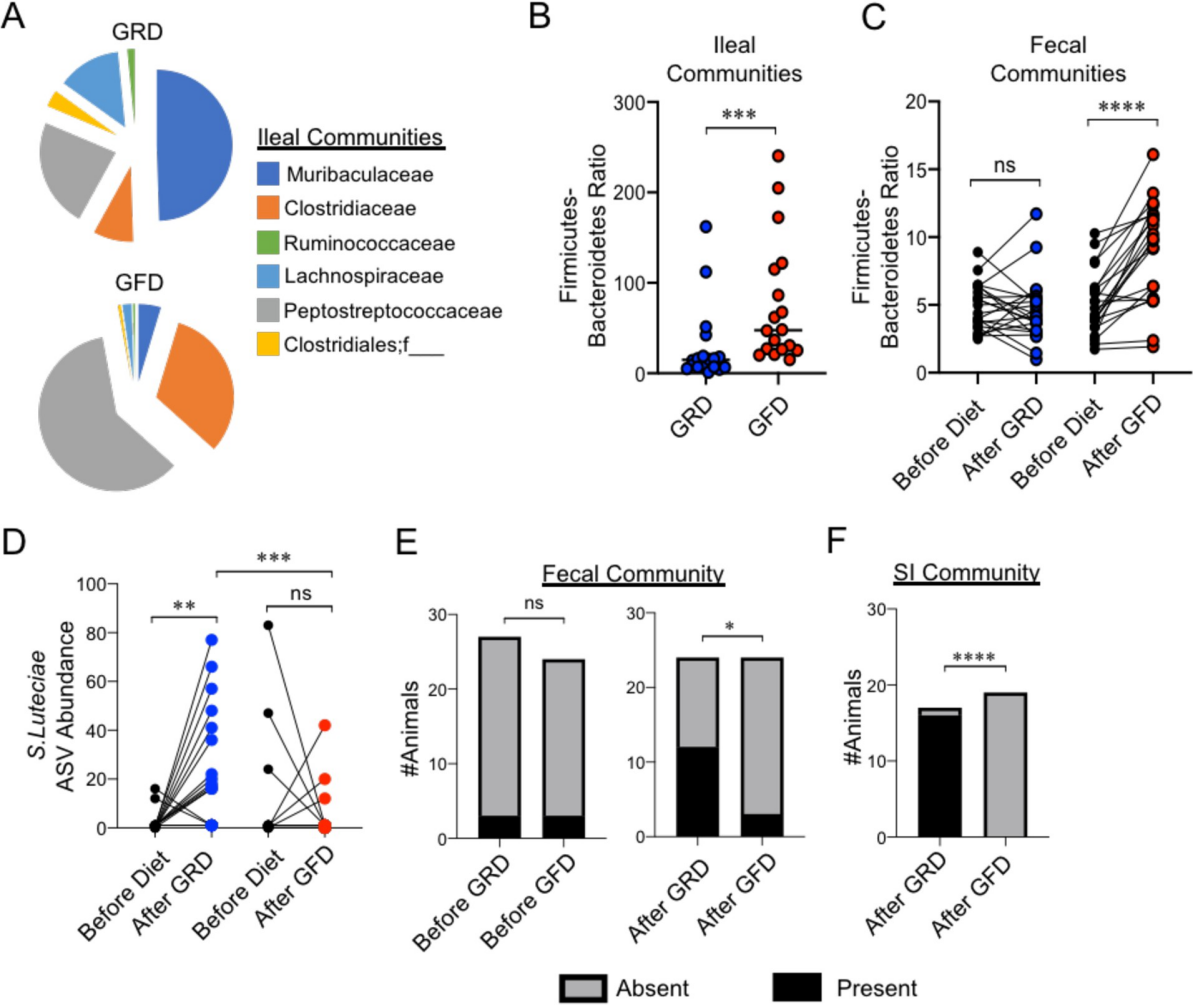
GFD exposure limits pathobiont colonization of the SI. **(A)** Pie charts depict the shifts in relative abundance of bacterial families whose abundance were significantly altered in the SI due to diet treatment. **(B)** SI community F-B ratios between GRD and GFD J_H_^-/-^ cohorts are shown. Two-tailed unpaired Student’s t-test; *** = p<0.001. **(C)** Fecal community F-B ratios between GRD and GFD J_H_^-/-^ cohorts are shown. Paired t-tests were used to compare how GRD or GFD exposure influenced fecal F-B ratios. A Mann-Whitney U test was used to compare how GRD and GFD exposure influenced SI F-B ratios. **(D)** *S*.*lutetiensis* ASV abundance in fecal communities before and after GRD or GFD exposure are shown. Paired t-tests were used to compare”before” vs. “after” groups, and an unpaired t-test was used to compare “after GRD” vs. “After GFD” groups. **(E)** Stacked bar-plots depicting the incidence of *S*.*lutetiensis* in fecal communities of GRD or GFD exposed mice are shown. Fisher’s exact test. **(F)** Stacked bar-plots depicting the incidence of *S*.*lutetiensis* in SI communities of GRD or GFD exposed mice are shown. Fisher’s exact test.

**Fig 5:**
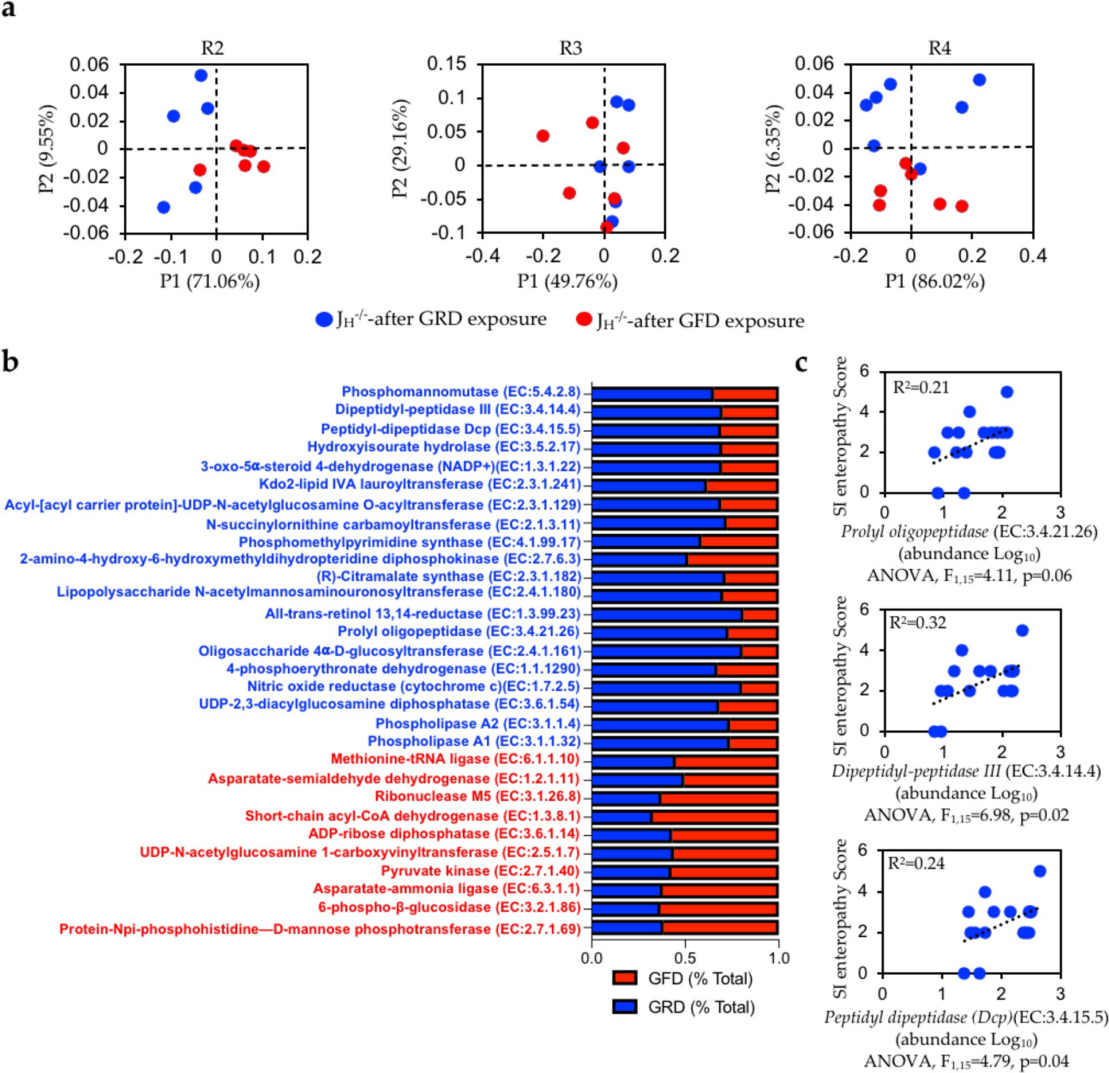
Dietary gluten may increase peptidase activity in the SI, which is positively correlated with SI enteropathy. **(A)** PcoA plots arranged by replicate experiment (based on Bray-Curtis analysis). PERMANOVA. **(B)** Differentially abundant inferred microbial genes are shown between GRD and GFD-fed J_H_^-/-^ mice. **(C)** Correlation analysis between the abundance of three microbial peptidase enzymes and SI enteropathy in GRD-fed mice are shown. One-way ANOVAs were used to analyze “gene x disease” correlations.

Statistical analyses of histology scores summarized in Figs 1, 2 and S2, were performed using Prism8.0 (Graphpad). A two-way ANOVA was used to determine the main effect of ’genotype’ or ’diet’ on GSE severity with degrees of freedom, F-statistics, and p-values reported in manuscript. A Fisher’s Exact test was used to statistically compare the incidence of disease between groups shown in Figs 1B and 2B. PERMANOVA results of β-diversity estimates shown in Fig 3A and summarized in Table 1 are based on Bray-Curtis distances and were generated using QIIME2.0 [26]. Bray-Curtis distances shown in Fig 3B and α-diversity estimates of ASV abundance (i.e. species richness) shown in Fig 3C were generated in QIIME2.0, with statistical analysis performed in PRISM8.0. Bray-Curtis distances were non-normal based on the Shapiro-Wilk test, so a Mann-Whitney U test was used for statistical hypothesis testing. Fecal ASV abundance was normally distributed with equal variance and an unpaired Student’s t-test was used for statistical hypothesis testing. SI ASV abundance was normally distributed with unequal variance and an unpaired Student’s t-test (with Welch’s correction for unequal variance) was used for statistical hypothesis testing. Results of linear regression analysis shown in Figs 3D, 4G, and 5C were performed in PRISM8.0 using a one-way ANOVA with degrees of freedom, F-statistics, and p-values reported in Figure. Bacterial families differentially enriched in SI communities and summarized in Fig 4A were identified using multiple hypothesis testing (unpaired two-tailed t-tests with Benjamini-Hochberg correction) of ASV sequence reads across observed Families. An FDR-cutoff of 0.1 was used). Firmicutes-Bacteroidetes ratios summarized in Fig 4B were non-normal and a Mann-Whitney U test was used for statistical hypothesis testing. A paired two-tailed t-test was used to compare the paired datasets shown in Fig 4C and 4D. A Fisher’s Exact test was used to statistically compare the incidence of *S*.*lutetiensis* between groups shown in Fig 4E and 4F. A Mann-Whitney U test was used to compare *S*.*lutetiensis* reads across experimental replicates in Supplementary Fig 3. PICRUSt results of β-diversity estimates shown in Fig 5A and summarized in Table 2 are based on Bray-Curtis distances and were generated using the PICRUSt2 QIIME2.0 plugin [27]. For PICRUSt analysis, differentially enriched genes in SI communities (summarized in Fig 5B) were identified using the DS-FDR QIIME2.0 plugin with an FDR-cutoff of <0.3.

**Table 1:** PERMANOVA results of β-diversity analyses (Bray-Curtis).

Dataset	Group1	Group2	Sample Size (n)	pseudo-F	p-value
Fecal Replicate 1	Before	GRD	18	5.52	0.001
Fecal Replicate 1	Before	GFD	18	16.65	0.001
Fecal Replicate 1	GRD	GFD	12	9.46	0.001
SI Replicate 1	-	-	-	-	-
Fecal Replicate 2	Before	GRD	16	7.47	0.001
Fecal Replicate 2	Before	GFD	17	15.52	0.001
Fecal Replicate 2	GRD	GFD	11	7.43	0.003
SI Replicate 2	GRD	GFD	11	13.32	0.002
Fecal Replicate 3*	Before	GRD	18	4.80	0.001
Fecal Replicate 3*	Before	GFD	18	7.40	0.001
Fecal Replicate 3*	GRD	GFD	12	1.65	0.14
SI Replicate 3*	GRD	GFD	12	1.48	0.16
Fecal Replicate 4	Before	GRD	20	6.18	0.001
Fecal Replicate 4	Before	GFD	19	7.64	0.003
Fecal Replicate 4	GRD	GFD	13	2.85	0.056
SI Replicate 4	GRD	GFD	13	3.21	0.032

PERMANOVA analyses based on 999 permutations.

*denotes J_H_^-/-^ mice co-housed with WT littermates until start of experiment.

**Table 2:** PICRUSt PERMANOVA results of β-diversity analyses (Bray-Curtis).

Dataset	Group1	Group2	Sample Size (n)	pseudo-F	p-value
SI Replicate 2	GRD	GFD	11	8.52	0.008
SI Replicate 3*	GRD	GFD	12	1.93	0.14
SI Replicate 4	GRD	GFD	13	0.55	0.47

PERMANOVA analyses based on 999 permutations.

*denotes J_H_^-/-^ mice co-housed with WT littermates until start of experiment.

## Results

### J_H_^-/-^ mice develop a gluten-sensitive SI enteropathy, which is a T cell-dependent phenotype

Previously, our group has demonstrated using the CD19^-/-^ mouse model that antibody-deficiency is associated with the development of a chronic SI enteropathy localized to the ileum of CD19^-/-^ mice [25]. Importantly, treatment with antibiotics or placing animals on a gluten-free diet alleviated disease, suggesting that defects in humoral immunity may have an important role in modulating the interaction between the microbiome and dietary gluten. CD19^-/-^ mice develop B cells but have defects in their activation and subsequent terminal differentiation into antibody secreting plasma cells, which leads to quantitative deficiencies in antibody titers in serum and mucosal secretions as well as qualitative defects in their response to specific antigens [25,28,29]. Therefore, to determine if absence of B cells results in a similar SI enteropathy to that observed in CD19^-/-^ mice we utilized a complementary mouse model of complete B cell deficiency (J_H_^-/-^ mice). First, blinded histological assessment using four features of SI enteropathy (extent of leukocytosis, extent of inflammation, villous blunting, and crypt hyperplasia) was performed on cohorts of WT C57BL/6 and J_H_^-/-^ mice bred and reared in our own mouse colony and maintained from weaning age on a gluten-rich diet (GRD)(Envigo, 2920X). This diet is the standard chow diet used to maintain all of the mice in our colony. Based on this analysis, we observed that J_H_^-/-^ mice developed a similar SI enteropathy to that observed in CD19^-/-^ mice (Fig 1A) characterized by significant increases in all four parameters of disease (two-way ANOVA, Fstat_1,48_ (main effect ’genotype’) = 118.3, p<0.0001) compared to WT animals (Fig 1B). Moreover, disease incidence (defined by a score of >1 in at least one of the four parameters of disease) was significantly increased in J_H_^-/-^ mice (8 of 8 (100%)) compared to WT animals (2 of 12 (17%))(Fisher’s exact test, p = 0.0007). Given previous observations using ileal transcriptomics that SI enteropathy in CD19^-/-^ mice was associated with enhanced T cell responses, we next compared SI enteropathy scores between CD19^-/-^, J_H_^-/-^, and RAG1^-/-^ mice to determine if the absence of T cells protected B cell deficient RAG1^-/-^ mice from disease. Consistent with this observation, RAG1^-/-^ mice developed minimal disease compared to T-cell-sufficient CD19^-/-^ (two-way ANOVA, Fstat_1,100_ (main effect ’genotype’) = 48.23, p<0.0001) and J_H_^-/-^ mice (two-way ANOVA, Fstat_1,56_ (main effect ’genotype’) = 54.84, p<0.0001)(Fig 1C). The incidence of disease was comparable between RAG1^-/-^ mice and CD19^-/-^ (Fisher’s exact test, p = 0.08) and J_H_^-/-^ mice (Fisher’s exact test, p = 0.21). This data lends further support to the notion that the SI enteropathy we have described in B-cell-deficient CD19^-/-^ and J_H_^-/-^ mice is driven by abnormal activation of T cells in the SILP.

### Exposure to a gluten-free diet ameliorates the severity of enteropathy in J_H_^-/-^ mice

To determine if the SI enteropathy we observed in J_H_^-/-^ mice was also sensitive to dietary gluten we performed four independent experiments where we randomly assigned 8–12 week old (male and female) littermate J_H_^-/-^ mice to one of two dietary treatments. Mice were either given *ad libitum* access to a GFD or a GRD from between 4–8 weeks of duration. Prior to commencing diet exposures, all mice had been maintained from weaning age on our standard GRD chow. At experimental endpoints, animals were euthanized and histological grading of SI enteropathy was performed by a blinded pathologist. Despite the fact that the extent of disease severity varied by experimental replicate, all four replicate experiments demonstrated a consistent trend in reduced disease severity in J_H_^-/-^ mice given access to a GFD (S2 Fig). Pooled analysis of SI enteropathy scores between GRD and GFD fed mice demonstrate that GFD significantly reduced disease severity in otherwise susceptible J_H_^-/-^ mice (two-way ANOVA, Fstat_1,220_ (main effect ’diet’) = 52.10, p<0.0001) (Fig 2A and 2B). These data indicate that J_H_^-/-^ mice develop a GSE phenotype similar to that which we have previously described for CD19^-/-^ mice.

### A GFD alters microbiota composition in J_H_^-/-^ mice

We hypothesized that if GSE is a microbiota-dependent phenotype in our model then we should observe consistent shifts in microbiota composition among experimental replicates that covary/correlate with SI enteropathy. To address this hypothesis we performed 16S rRNA gene sequencing to characterize fecal as well as SI (ileal) microbial community composition in GRD- and GFD-fed J_H_^-/-^ mice. In general and as expected, β-diversity analyses (based on Bray-Curtis distances) across replicate experiments demonstrated that diet treatment significantly altered fecal and SI bacterial communities (Fig 3A) (Table 1). Non-lethal sampling of fecal communities allowed us to analyze divergence in microbiota composition as a function of diet. The divergence in community composition after diet treatment was greater in mice that were placed on a GFD versus those that were kept on a GRD (Fig 3B). Pooled analysis of ⍺-diversity estimates (i.e. observed species (a.k.a. ’ASVs’)) across replicates demonstrated that GFD-fed J_H_^-/-^ mice had similar species richness in fecal communities to that observed GRD-fed mice, but had significantly fewer species in SI communities (Fig 3C). Additionally, we observed that SI species diversity is positively correlated with disease severity (linear regression: R^2^ = 0.43, ANOVA, Fstat_1,15_ = 11.29, p = 0.004) (Fig 3D), whereas fecal species diversity was not (linear regression: R^2^ = 0.03, ANOVA, Fstat_1,21_ = 0.6340, p = 0.43). Because of this observation we chose to focus the remainder of our 16S analyses primarily on SI-resident microbial communities.

### GFD exposure limits pathobiont colonization of the SI

In the SI, taxonomic analysis of microbial communities revealed several microbial phenotypes that were influenced by diet. From a broad taxonomic perspective we found that the abundance of bacteria within six bacterial families were differentially enriched in SI communities as a consequence of diet exposure (Fig 4A) (S1 Table). Within the phylum Bacteroidetes, GFD-fed J_H_^-/-^ mice had reduced abundance of bacteria within the family *Muribaculaceae* (formerly S24-7) [30] and increased abundance of bacteria within the *Peptostreptococcaceae* family. Within the phylum Firmicutes, GFD-fed J_H_^-/-^ mice had increased abundance of bacteria within the family *Clostridiaceae* and decreased abundance of bacteria within an unknown Clostridia family as well as bacteria within the families *Ruminococcaceae* and *Lachnospiraceae*. Shifts in the relative abundance of these bacterial families were not predictive of disease severity (not shown). We also observed that the Firmicutes-Bacteroidetes ratio was increased in the SI of GFD-fed J_H_^-/-^mice (Fig 4B). This was a specific effect caused by GFD exposure as the fecal Firmicutes-Bacteroidetes ratio was consistent between pre- and post-exposure timepoints in GRD-fed J_H_^-/-^ mice but was increased post-GFD exposure (Fig 4C). Despite the observation that SI Firmicutes-Bacteroidetes ratios were significantly affected by diet, this phenotype was not predictive of disease severity in GRD-fed J_H_^-/-^ mice (linear regression: R^2^ = 0.002, ANOVA, Fstat_1,16_ = 0.03315, p = 0.86). At the level of ASV, we observed that diet had a significant effect on the relative abundance of several ASVs in the SI (S2 Table). However for replicate #3, none of these ASVs achieved statistical significance after DS-FDR p-value adjustment. Moreover, none of the differentially abundant ASVs were consistently observed across experimental replicates, with the exception of one. Interestingly, across four independent replicate experiments we find a consistent effect of GFD exposure on both the incidence and relative abundance of the pathobiont, *Streptococcus lutetiensis* (S3 Fig). Overall, in fecal communities; we observed that *S*. *lutetiensis* abundance is significantly expanded in mice maintained on a GRD, but not GFD, during the course of our experiments (Fig 4D). Moreover, while the fecal incidence of *S*.*lutentiensis* was consistent between J_H_^-/-^ mouse cohorts prior to diet treatment (Fig 4D, left panel), it was significantly and specifically reduced in the fecal communities of mice that were exposed to a GFD (Fig 4D, right panel). Finally, in SI communities, we observed that GFD exposure completely eliminated the presence of this pathobiont (Fig 4E) (S3 Fig). However, the abundance of this pathobiont was not predictive of disease severity in GRD-fed J_H_^-/-^ mice (linear regression: R^2^ = 0.09, ANOVA, Fstat_1,16_ = 1.582, p = 0.23).

### Dietary gluten may increase peptidase activity in the SI, which is positively correlated with SI enteropathy

Given the observed changes in microbiota composition in the SI of GRD and GFD treated mice, we next wanted to gain insight into what metabolic pathways and enzymes were differently expressed. To do this, we utilized PICRUSt2; a method for inferring microbial gene content from 16S datasets. Pathway analysis did not reveal consistent differences in microbial functions among our three replicate experiments (Fig 5A) (Table 2). However, several microbial genes were differentially abundant between GRD and GFD treated J_H_^-/-^ mice (Fig 5B) (S3 Table). In general, GFD-fed J_H_^-/-^ mice were enriched in several genes associated with carbohydrate metabolism, while GRD-fed mice were enriched in several genes associated with lipopolysaccharide (LPS) biosynthesis, and phospholipid and protein metabolism. More specifically, J_H_^-/-^ mice administered GRD chow were enriched in three genes that were identified as microbial peptidases (*Dipeptidyl-peptidase III* (EC:3.4.14.4), *Peptidyl-Dipeptidase Dcp* (EC:3.4.15.5), *Prolyl oligopeptidase* (EC:3.4.21.26)), two microbial phospholipases (*Phospholipase A1* (EC:3.1.1.32), *Phospholipase A2* (EC:3.1.1.4)), and three genes associated with LPS biosynthesis (*Lipopolysaccharide N acetylmannosaminouronosyltransferase* (EC:2.4.1.180), *Kdo2 lipid IVA lauroyltransferase* (EC:2.3.1.241), *acyl-acp-udp-n-acetylglucosamine o-acyltransferase* (EC:2.3.1.129)). J_H_^-/-^ mice administered a GFD were enriched for several microbial genes associated with carbohydrate metabolism (*pyruvate kinase* (EC:2.7.1.40), *6 phospho-β glucosidase* (EC:3.2.1.86), *UDP-N acetyl glucosamine 1 carboxyvinyltransferase* (EC:2.5.1.7)) and bacterial protein synthesis (*Protein Npi-phosphohistidine-D mannose phosphotransferase* (EC:2.7.1.69), *aspartate ammonia ligase* (EC:6.3.1.1)). In addition to covarying with disease severity, correlation analysis also revealed a significant positive correlation between GSE severity and the abundance of all three bacterial peptidase genes in GRD-fed J_H_^-/-^ mice (Fig 5C).

## Discussion

In a previous study using a CD19^-/-^ mouse model of humoral immunodeficiency we were able to describe a novel disease phenotype restricted to the SI of mice that appeared superficially to resemble CeD in humans. This assessment was based on several immunological/physiological features shared with CeD patients [25]; the most significant being resolution of GSE when exposure to dietary gluten is eliminated. However, since these mice are not transgenic for any of the HLA-DQ risk alleles they are not a model of HLA-dependent GSE so we refrained from calling them a model of CeD. Despite this limitation to the model, they appear to develop a similar disease phenotype. In the current study we extend upon these observations to demonstrate that a gluten-sensitive SI-specific enteropathy also develops in J_H_^-/-^ mice, which completely lack B cells. The results from both of these studies suggest that a diminished humoral immune response enhances sensitivity to gluten. This is not without precedent, as humoral immunodeficiency has previously been shown to enhance immune responses to other dietary antigens in both mouse models [31–33] as well as humans [34–37]. From these observations we sought to determine how exposure to a gluten-free diet influences the microbiota and report several interesting findings. Notably, we observe an increase in the Firmicutes-Bacteroidetes ratio in GFD-fed J_H_^-/-^ mice; a commonly used metric of dysbiosis. We also find that species diversity is reduced in the SI of GFD-fed J_H_^-/-^ mice and that microbial diversity in the SI is positively correlated with disease severity in GRD-fed J_H_^-/-^ mice. Reduced microbial diversity in the SI of GFD-fed J_H_^-/-^ mice was associated with the complete elimination of a known pathobiont *S*.*lutetiensis*. Finally, we report that the abundance of several bacterial peptidase genes are reduced in the SI of GFD-fed J_H_^-/-^ mice, and that the abundance of these genes are positively correlated with the severity of SI enteropathy.

An association between humoral immunity and gluten sensitivity has long been appreciated and is widely exploited in the diagnosis of CeD in humans. For example, anti-gliadin, anti-tTG, and anti-EMA ELISAs are routinely used as non-invasive methods to confirm suspicion of an underlying gluten sensitivity by physicians. In fact, the early association of the presence of anti-tTG antibodies with celiac disease [12] is the primary reason why celiac disease is considered an auto-immune disorder, despite little evidence to support that anti-tTG, anti-EMA, or AGA antibodies play a role in pathogenesis. Indeed, as mentioned above, CeD patients are 10–15 times more likely to have an associated IgA deficiency [14], implying that IgA may play a protective role. A more recent study also suggest that IgG antibodies generated against gliadin and tTG may also play a protective role. For example, a major difference between CeD and NCGS is the explicit development of a GSE in the former [7,38]. Interestingly, a recent study found that both patient cohorts developed similar titers of serum IgG antibodies reactive to gliadin and tTG, but significantly differed in their development of specific IgG subclasses [39]. NCGS patients were found to be enriched for IgG4 antibodies (an IgG subclass with weak immuno-stimulatory properties) while CeD patients were enriched for IgG1 and IgG3 subclasses, which are highly immuno-stimulatory. The authors speculate that this bias could be an underlying explanation for the difference in disease manifestations between CeD and NCGS patients. More importantly, they highlight the importance of considering the functions of antibodies generated in response to gluten, rather than their simple presence. For example, some anti-tTG antibodies may decrease the enzymatic activity of tTG [40], and the development of neutralizing antibodies against gliadin [41] suggests that some AGAs generated during CeD may have a protective effect. Explicitly defining the role anti-tTG and anti-gliadin antibodies play in GSE pathogenesis *in vivo*, requires the use of mouse models where these responses can be modelled and their physiological effects quantified.

Currently, data from a number of mouse models also provide equivocal support for the hypothesis that antibody responses play a pathogenic role in GSE. First, a study by Freitag et al. demonstrated that athymic (i.e. ’nude’) mice that were fed a GRD and administered gluten-reactive T cells failed to develop histological changes associated with the elevation of anti-gliadin IgG and IgA [42]. Additionally, the elevation in anti-tTG IgG and IgA was also associated with normal histological findings in the DQ8.D^d^-IL-15Tg-Aβ^0^ mouse model [42,43]. Two mouse models have previously been shown to develop an SI-specific GSE. In the same study mentioned above, Frietag et al. demonstrated that athymic mice, which do not possess T cells but have functional B cells, do not develop GSE whereas RAG1^-/-^ mice (which do not possess functional T cells or B cells) do [42], which implies that B cells may play a tolerizing role to gluten-reactive T cells. In contrast, a study published this year by Lejeune et al. has demonstrated using a recently described transgenic mouse model of CeD (the DQ8.D^d^-villin-IL-15Tg mouse model) [44] that GSE is moderately reduced when mice are administered rituximab (an anti-CD20 monoclonal antibody) or crossed to B-cell-deficient μMT^-/-^ mice [45]. However, despite titers of AGA antibodies being reduced in these two experiments, plasma cell abundance in the gut is maintained to normal levels so the physiological role of antibodies in GSE pathogenesis is not adequately modelled. Here, we have provided data from a second mouse model of humoral immunodeficiency, that B cells may have a tolerizing effect during exposure to dietary gluten antigens.

Numerous studies have associated CeD in humans with alterations to microbiota composition [46], but to date, no "core celiac microbiota" has been identified. One could infer from this that either the microbiota is irrelevant, or that multiple different bacterial species are capable of influencing GSE pathogenesis. Evidence derived from animal models heavily favors the latter interpretation. In non-ruminant mammals (including humans and mice), gluten is a relatively indigestible complex of proteins, and under normal conditions most gluten catabolism is performed by microbes in the large intestine. These microbes contain a variety of peptidases capable of hydrolyzing the glutamine-proline rich residues of gliadin. Bacterial hydrolysis of peptides may render them less or more likely to initiate an immune response (depending on the bacterial species) [2,46,47]. Importantly, the presence of a microbiota, and shifts in its composition, have been shown to directly influence GSE pathogenesis. Using the diabetic NOD-Aβ^0-^DQ8 mouse model of GSE, a model of GSE dependent on gliadin/cholera toxin pre-sensitization [48], Galipeau et. al demonstrated that germfree NOD-Aβ^0-^DQ8 mice developed a GSE that could be ameliorated by colonizing animals with a microbiota derived from specific-pathogen-free (SPF) mice [49]. In contrast, germfree mice colonized with a microbiota that contained pathobionts were not protected from GSE. In humans, the dominant group of bacteria in the SI thought to play a role in gluten catabolism include species representing several genera within the Firmicutes phylum including *Lactobacillus*, *Streptococcus*, *Staphylococcus*, and *Clostridia* [46,50]. However, species from other Phyla may also contribute and in fact the best current evidence to support that specific features of dysbiosis observed in celiac patients can be a predisposing factor for GSE comes from them. For example, Caminero et al. demonstrated using *in vitro/ex vivo* techniques that the opportunistic pathogen *Pseudomonas aeruginosa* (Proteobacteria Phylum), found to be enriched in the SI of a cohort of celiac patients, was able to generate gluten peptides with increased capacity for translocation into the lamina propria as well as enhanced capacity to stimulate an inflammatory T cell response *in vitro* [2]. In contrast, a Lactobacillus isolate derived from healthy patients reversed these effects. These are important observations for two reasons. First, it is the only demonstration to our knowledge of the differential capacity of commensal bacteria to regulate gluten immunogenicity. Second, it provides evidence that commensal bacteria may influence gluten sensitivity by enhancing both the generation of immunogenic peptides and their translocation into the underlying lamina propria. More recently, using an elegant series of experiments Petersen et al. were able to demonstrate that multiple different bacterial species (including *P*.*aeruginosa*, as well as another *Pseudomonas* species, and several other unrelated species from the Firmicutes and Bacteroidetes Phyla) express their own peptides that mimic the structure of gliadin peptides, and that these peptides can stimulate gliadin-reactive T cells [51]. This is a crucial observation because it demonstrates a secondary pathway through which the microbiota can drive GSE independent of modification of gluten antigens. Previous results from our studies in CD19^-/-^ mice [25] and our current study in J_H_^-/-^ mice indicate that the microbiota may also play a role in GSE pathogenesis in mice. First, we found that the GSE that develops in CD19^-/-^ mice can be alleviated by administering antibiotics to animals. Second, in this study we found that microbial peptidase gene abundance was positively correlated with disease severity in GRD-fed J_H_^-/-^ mice. Thus, accumulating evidence clearly supports that the microbiota can play an important role in GSE pathogenesis in multiple ways. Mouse models of GSE will be instrumental for dissecting these interactions.

Diet is a major regulator of microbiota composition and function, and expectedly, exposure to a GFD is associated with shifts in microbiota composition [52]. In the SI microbial community, we also find that exposure to a GFD results in shifts in composition; primarily increased Firmicutes-Bacteroidetes ratios, decreased abundance of ASVs belonging to the *Muribaculaceae* Family and increased abundance of ASVs belonging to the *Clostridiaceae* Family. GFD exposure also results in shifts in the relative abundance of specific ASVs in the SI, though none of these ASVs were consistently observed across replicates (with the exception of one (more below)). This lack of consistency in the effect of GFD on microbiota composition is largely what has been observed in humans [52] and is not unexpected. For example, it is well-known that the microbiota is tremendously variable among even healthy individuals. Additionally, studies performed to look at the relationship between CeD and microbiota composition vary widely in terms of patient demographics, sampling strategies, and methods employed to characterize microbiota composition. Despite these limitations, in studies that have identified shifts in microbiota composition in CeD patients, the overwhelming majority of them have observed increases in the abundance of a wide array of pathobionts (or overt pathogens) including *Escherichia coli*, *Prevotella sp*., *Bacteroides dorei*, *Parabacteroies distansonis*, *Prevotella melaninogenica*, *Klebsiella oxytoca*, *Staphylococcus epidermidis*, *Enterobacteriaceae* members, *Streptococcus sanguinus*, *Rothia mucilaginosa*, *Leptotrichia* sp., *Veilonella parvula*, *Stenotrophomonas maltophilia*, and *P*. *aeruginosa* [46,50]. Two studies in mice support that pathobionts play a role in GSE pathogenesis. First, as mentioned above, Caminero et al. demonstrated that *P*.*aeruginosa* isolated from CeD patients enhanced production of immunogenic gliadin peptides, translocation of gliadin peptides across the gut barrier, and T cell responses [2]. Second, Galipeau et al. demonstrated that the protective effect of an SPF microbiota on GSE pathogenesis could be reversed by colonizing SPF NOD-Aβ^0-^DQ8 mice with a pathogenic *E*.*coli* strain isolated from celiac patients [49]. Importantly, CeD patients have increased susceptibility to colonization by bacterial pathogens (including *Stretococcus* species) [50,53,54].

Bacteria within the *Streptococcus* genus are dominant members of the small intestinal microbiome of humans [55]. *Streptococcus* species within the Viridans *Streptococcus* group, *Streptococcus anginosus* group, and *Streptococcus bovis* group have been isolated from human feces and shown to participate in gluten metabolism [50,56,57]. Numerous members of all of these groups are opportunistic pathogens. Due to the inadequacy of 16S sequencing to accurately identify ASVs down to species and a lack of ability to discriminate between closely-related members of various *Streptococcus* groups; *’S*.*lutetiensis’* is likely a misnomer. In fact, a BLAST search using representative sequences for *S*. *lutetiensis* derived from our sequence data resulted in several hits (each with a percentage identity of 100%) coming *from S*. *equinus*, *S*. *infantarius*, *and Streptococcus vicugnae*; each members of the *S*.*bovis* group. Despite this lack of resolution, our data clearly demonstrates that exposure to a GFD consistently leads to the elimination of this group of bacteria from the SI of J_H_^-/-^ mice. This could prohibit GSE in several ways. The microbiota may directly contribute to gluten sensitivity by enhancing the bioavailability of immunogenic gluten peptides through modification of gluten antigens, or indirectly by increasing gut permeability and the translocation of immunogenic gluten peptides into the underlying *lamina propria* where they can stimulate gluten-reactive T cell subsets. The *S*.*bovis* group may be able to perform both functions as suggested by work done in livestock. As noted above the *S*.*bovis* group is able to metabolize gluten, but may also have the capacity to make it more immunogenic. For example, bacterial transglutaminase, an enzyme broadly conserved across bacteria that has long-been used in the food industry to make food more palatable, is known to cross-link gliadin peptides and enhance their immungenicity [58,59]. *Streptococcus suis*, an important pathogen of pigs, possesses a mTG that serves as a virulence factor by inhibiting phagocytosis [60]. To our knowledge, whether mTGs isolated from *Streptococcus* species enhances gluten immunogenicity has not been addressed. Interestingly, in livestock, an acute shift to a wheat-based diet is known to lead to lactic acidosis caused by overgrowth of lactic-acid-producing bacteria; primarily *Lactobacillus* and *S*.*bovis* group members [61]. Lactic acidosis results in degradation to the mucosal lining in the gut and a decrease in the ability of livestock to absorb nutrients and grow. *Streptococcus* species also express a variety of toxins capable of disrupting epithelial barriers [62]. Our results indicate that a diet rich in gluten promotes colonization of the SI by *S*.*lutetiensis* which may enhance SI permeability and passive translocation of immunogenic gluten peptides into the underlying *lamina propria*. Given the prevalence of *Streptococcus* in the SI of humans, the ability of members of this group to metabolize/modify gluten antigens, and their capacity to enhance exposure of the mucosal immune response to dietary antigens, further examination of this bacterial clade in GSE pathogenesis is warranted.

In addition to microbial taxonomic composition, we also sought to understand what microbial functions were differentially-enriched by diet treatment and how they related to the severity of GSE. We found that the abundance of several specific microbial enzymes were differentially-enriched between GRD- and GFD-fed J_H_^-/-^ mice. We found that two bacterial phospholipases (A1 and A2) were enriched in GRD-fed mice. Bacterial phospholipases can act as virulence factors that aid in adhesion to gut epithelial cells, destabilization of tight junctions, and enhanced intestinal permeability [63]. Thus, increased microbial phospholipase activity could enhance gluten sensitivity by destabilizing epithelial integrity and promoting the translocation of immunogenic gluten peptides into the underlying *lamina propria*. We also found that mice fed a GRD also had higher abundance of three microbial peptidase genes (*Dipeptidyl-peptidase III*, *Peptidyl-Dipeptidase Dcp*, *Prolyl oligopeptidase*), suggesting that microbial modifications to gluten antigens may be enhanced in these animals. That this may exacerbate GSE in our mice is evidenced by the observed significant positive relationship between peptidase gene abundance and GSE severity in GRD-fed J_H_^-/-^ mice. Dipeptidyl-peptidase III and Prolyl Oligopeptidase are members within the M4 and S9 families respectively and have the capacity to degrade the immunogenic portion of gluten [47]. For this reason, harnessing bacterial peptidases as potential treatments for celiac disease has been considered. However, it must be noted that different bacterial species possess their own versions of these enzymes, and some bacterial species possess peptidase enzymes that can expose immunogenic gluten epitopes. For example, *elastase B* is a metallopeptidase expressed by *Pseudomonas aeruginos*a that can be used to degrade mucins and surfactant proteins^2^ and cleaves gluten in such a way that multiple immunogenic peptides are formed [64].

There are several limitations to our model. First, and most importantly, we have been careful not to label this a model of CeD. These mice do not model HLA-dependent GSE pathogenesis since they do not carry disease-relevant HLA alleles. Also, while our phenotyping data demonstrates that the GSE that develops in J_H_^-/-^ (and previously in CD19^-/-^) mice is a T-cell-dependent phenomenon, we have not demonstrated that this enteropathy is an antigen-specific response to gluten peptides (though the recent findings by Petersen et al. demonstrate that this may not be a requirement for the initiation of gluten sensitivity [51]). We simply refer to these mice as a model of gluten sensitivity driven by an immunopathological response occurring in the presence of dietary gluten. A second limitation to our models is that, like all previous studies in other murine models, we have not explicitly considered the role of antibodies in GSE pathogenesis. This is an outstanding question in the field of GSE research, and one that requires an animal model to address. Adoptive transfer models in J_H_^-/-^ mice will allow us to answer this long-standing question. Finally, our experiments are also only correlative in nature, and future studies will be needed to empirically define the contribution of the microbiota in GSE pathogenesis. However, we have now identified a specific pathobiont that can be isolated and assessed for its ability to satisfy Koch’s postulates, with ongoing experiments in our lab focused on this effort.

## Conclusions

Collectively, the results of the experiments we have described in this study emphasize the need to further dissect the influence of humoral immunity on GSE pathogenesis. Taken as a whole, we have now demonstrated in two murine models of B cell deficiency (CD19^-/-^ and J_H_^-/-^ mice) that humoral immunity may actually tolerize individuals to gluten antigens, which is contrary to what is generally assumed. How B cells do this is a matter for future work, but could operate through the production of antibodies that neutralize pathogenic gluten epitopes, restrict colonization of the SI by gluten-metabolizing bacteria, or restrict colonization of the SI by pathogens that enhance translocation of dietary antigens into the lamina propria. It is also possible that B cells may suppress activation of gluten-reactive T cells through an antibody-independent mechanism like IL-10 secretion. Finally, the most significant contribution of this work is to highlight another mouse model of B cell deficiency that may be useful for studying the interaction between B cells, the microbiota, and dietary gluten. Such models are lacking and would be of significant benefit to basic research on gluten sensitivity because of the tractability of the laboratory mouse.

## Supporting information

S1 Fig ⍺-rarefaction (i.e. collector’s) curves demonstrating equal sampling of microbial diversity between treatment groups. (a and b) For analysis of both (a) fecal and (b) SI bacterial communities, ASV tables were rarified down to an equal sampling depth of 17649 sequences per sample. Sampling of bacterial diversity was equivalent among treatment groups as demonstrated by the consistent plateau in observed species as a function of sampling depth. The results of all statistical analyses reported in this manuscript were based on abundance data derived from rarified feature tables.
(PDF)

S2 Fig Exposure to a GFD results in a consistent reduction in disease severity across four experimental replicates. (a-d) SI enteropathy scores and the incidence of detectable disease phenotypes are shown for each experimental replicate. Two-way ANOVAs were used to compare the effects of diet on the severity of SI enteropathy. (a) Two-way ANOVA, Fstat_1,72_ = 24.32, p<0.0001. (b) Two-way ANOVA, Fstat_1,40_ = 5.213, p = 0.0278. (c) Two-way ANOVA, Fstat_1,40_ = 3.913, p = 0.0548 (d) Two-way ANOVA, Fstat_1,44_ = 17.51, p = 0.0001.
(PDF)

S3 Fig *S*.*lutetiensis* abundance/incidence is repeatably regulated by exposure to a gluten free diet. The relative abundance of *S*.*lutetiensis* in fecal and SI-resident communities are shown for each of the experimental replicates performed in this study. Mann-Whitney U test; ns = non-significant, ** = p<01.
(PDF)

S4 Fig Bacterial peptidase abundance is influenced by exposure to GRD. The relative abundance of three bacterial peptidase genes across experimental replicates are shown. Mann-Whitney U test; ns = non-significant, * = p<0.05, ** = p<01, **** = P<0.0001.
(PDF)

S1 Table SI bacterial families. A Microsoft Excel table is provided that lists the relative abundance of AVS reads by bacterial family across samples is provided. Results of FDR-adjusted multiple hypothesis testing is also provided.
(XLSX)

S2 Table DS-FDR results. A Microsoft Excel table is provided that summarizes the results of DS-FDR statistical hypothesis testing to identify significantly differentially enriched ASVs.
(XLSX)

S3 Table PICRUSt predicted EC genes. A Microsoft Excel table is provided that lists significantly differentially enriched predicted microbial genes based on PICRUSt analysis.
(XLSX)
