# Supplementary material for: Gluten-free diet exposure prohibits pathobiont expansion and gluten sensitive enteropathy in B cell deficient JH-/- mice
Source: PLoS One. 2022 Mar 24;17(3):e0264977. doi: 10.1371/journal.pone.0264977 (PMC8946719; doi:10.1371/journal.pone.0264977)
Supplement: S1 Fig — (a and b) For analysis of both (a) fecal and (b) SI bacterial communities, ASV tables were rarified down to an equal sampling depth of 17649 sequences per sample. Sampling of bacterial diversity was equivalent among treatment groups as demonstrated by the consistent plateau in observed species as a function of sampling depth. The results of all statistical analyses reported in this manuscript were based on abundance data derived from rarified feature tables. (PDF) [file pone.0264977.s001.pdf]

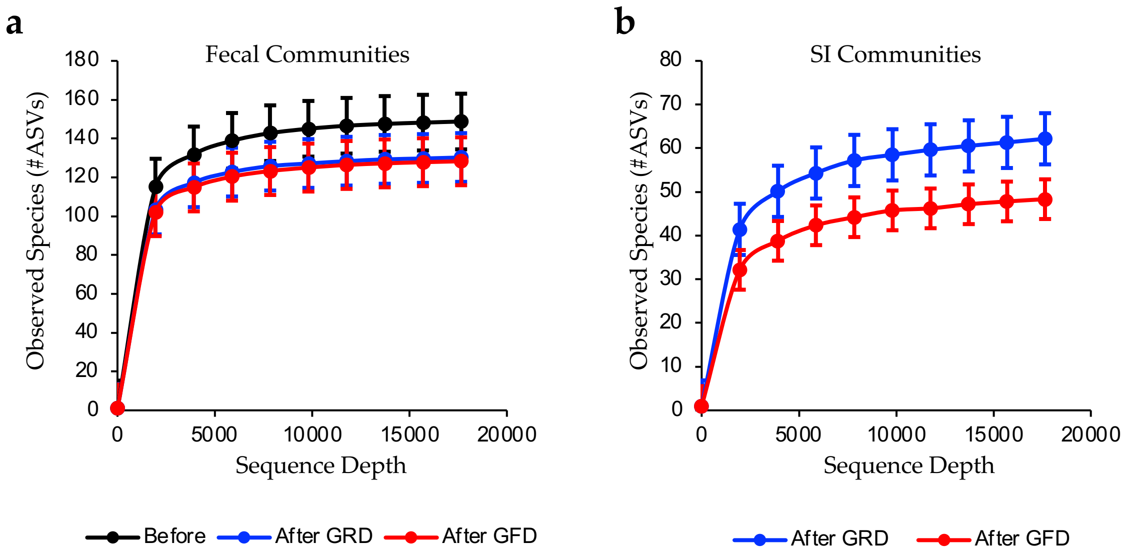

Fig S1. α-rarefaction (i.e. collector's) curves demonstrating equal sampling of microbial diversity between treatment groups. (a and b) For analysis of both (a) fecal and (b) SI bacterial communities, ASV tables were rarified down to an equal sampling depth of 17649 sequences per sample. Sampling of bacterial diversity was equivalent among treatment groups as demonstrated by the consistent plateau in observed species as a function of sampling depth. The results of all statistical analyses reported in this manuscript were based on abundance data derived from rarified feature tables.
