# Supplementary material for: Gluten-free diet exposure prohibits pathobiont expansion and gluten sensitive enteropathy in B cell deficient JH-/- mice
Source: PLoS One. 2022 Mar 24;17(3):e0264977. doi: 10.1371/journal.pone.0264977 (PMC8946719; doi:10.1371/journal.pone.0264977)
Supplement: S2 Fig — (a-d) SI enteropathy scores and the incidence of detectable disease phenotypes are shown for each experimental replicate. Two-way ANOVAs were used to compare the effects of diet on the severity of SI enteropathy. (a) Two-way ANOVA, Fstat1,72 = 24.32, p<0.0001. (b) Two-way ANOVA, Fstat1,40 = 5.213, p = 0.0278. (c) Two-way ANOVA, Fstat1,40 = 3.913, p = 0.0548 (d) Two-way ANOVA, Fstat1,44 = 17.51, p = 0.0001. (PDF) [file pone.0264977.s002.pdf]

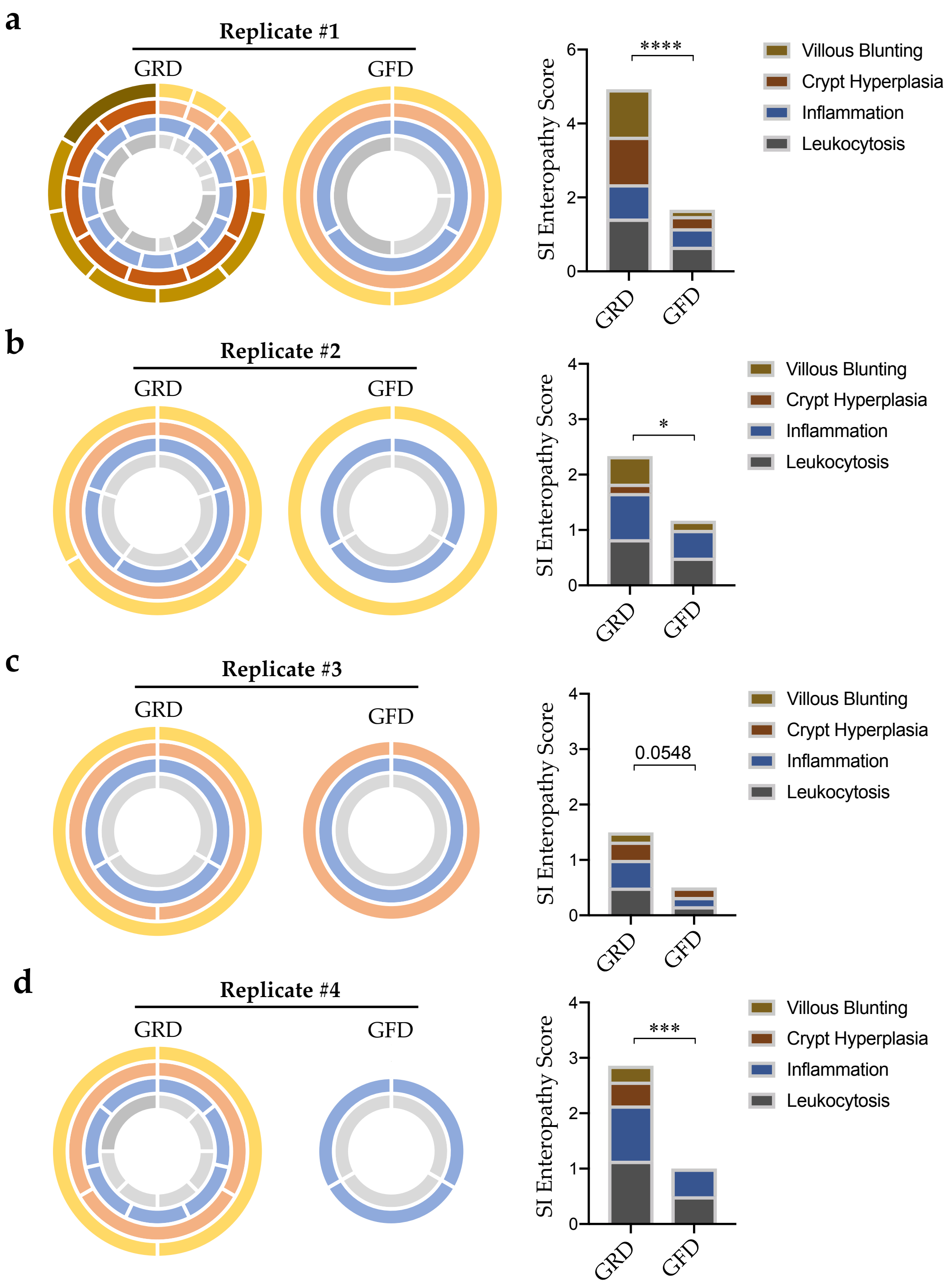

Fig S2. Exposure to a GFD results in a consistent reduction in disease severity across four experimental replicates. (a-d) SI enteropathy scores and the incidence of detectable disease phenotypes are shown for each experimental replicate. Two-way ANOVAs were used to compare the effects of diet on the severity of SI enteropathy. (a) Two-way ANOVA, $Fstat_{1,72}$=24.32, p<0.0001. (b) Two-way ANOVA, $Fstat_{1,40}$=5.213, p=0.0278. (c) Two-way ANOVA, $Fstat_{1,40}$=3.913, p=0.0548 (d) Two-way ANOVA, $Fstat_{1,44}$=17.51, p=0.0001.
