# Supplementary material for: Gluten-free diet exposure prohibits pathobiont expansion and gluten sensitive enteropathy in B cell deficient JH-/- mice
Source: PLoS One. 2022 Mar 24;17(3):e0264977. doi: 10.1371/journal.pone.0264977 (PMC8946719; doi:10.1371/journal.pone.0264977)
Supplement: S3 Fig — The relative abundance of S.lutetiensis in fecal and SI-resident communities are shown for each of the experimental replicates performed in this study. Mann-Whitney U test; ns = non-significant, ** = p<01. (PDF) [file pone.0264977.s003.pdf]

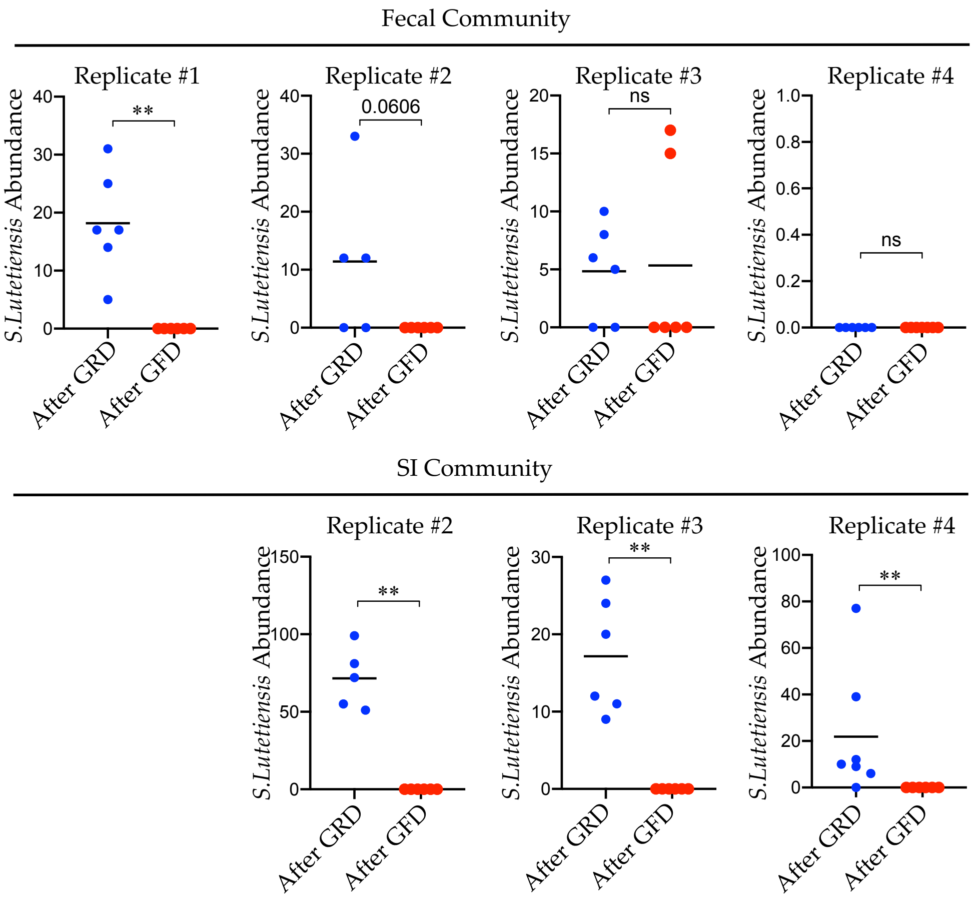

Fig S3. *S.lutetiensis* abundance/incidence is repeatably regulated by exposure to a gluten free diet. The relative abundance of *S.lutetiensis* in fecal and SI-resident communities are shown for each of the experimental replicates performed in this study. Mann-Whitney U test; ns=non-significant, **=p<01.
