# Supplementary material for: Gluten-free diet exposure prohibits pathobiont expansion and gluten sensitive enteropathy in B cell deficient JH-/- mice
Source: PLoS One. 2022 Mar 24;17(3):e0264977. doi: 10.1371/journal.pone.0264977 (PMC8946719; doi:10.1371/journal.pone.0264977)
Supplement: S4 Fig — The relative abundance of three bacterial peptidase genes across experimental replicates are shown. Mann-Whitney U test; ns = non-significant, * = p<0.05, ** = p<01, **** = P<0.0001. (PDF) [file pone.0264977.s004.pdf]

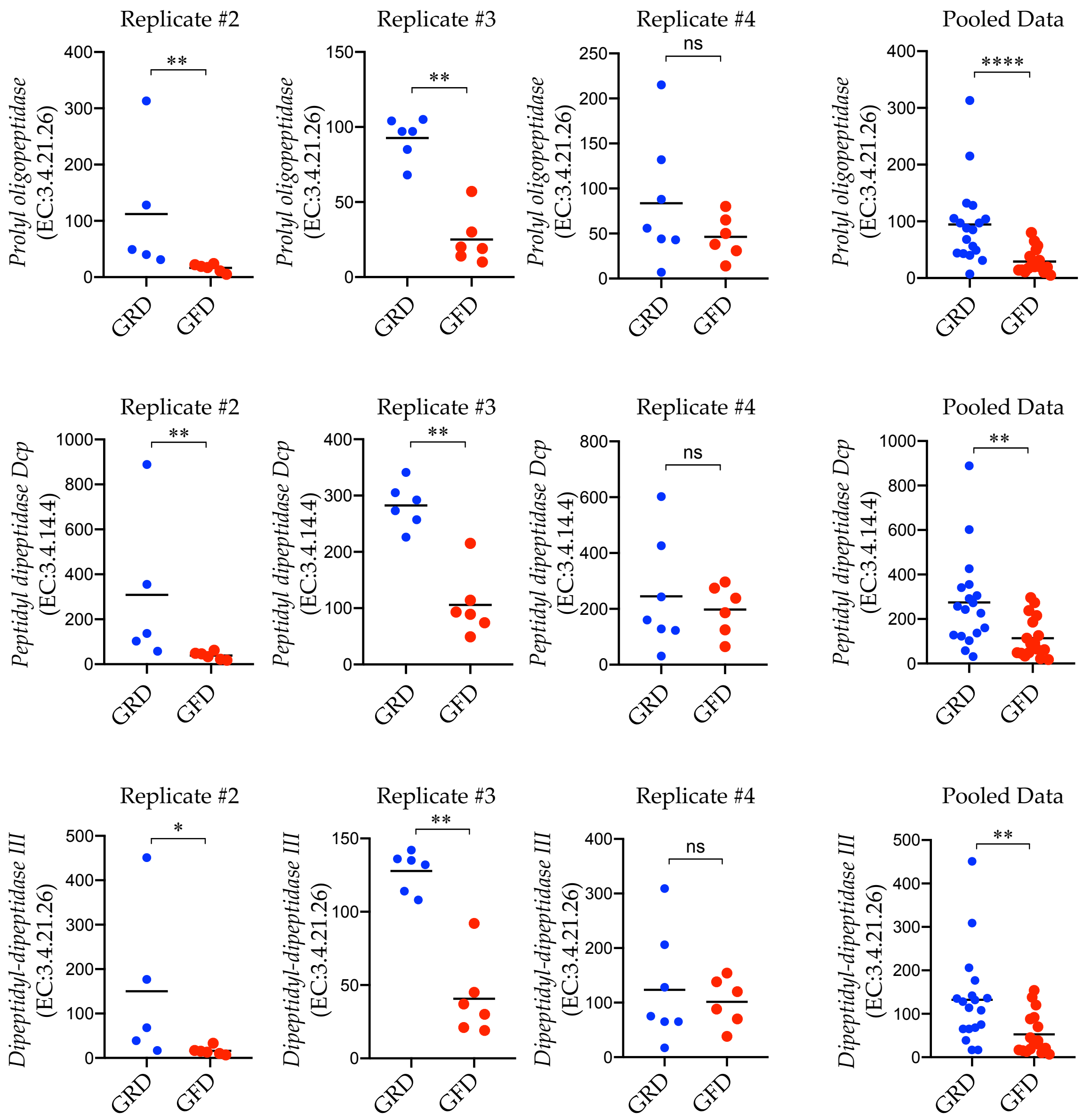

Fig S4. Bacterial peptidase abundance is influenced by exposure to GRD. The relative abundance of three bacterial peptidase genes across experimental replicates are shown. Mann-Whitney U test; ns=non-significant, *=p<0.05, **=p<01, ****=P<0.0001.
